# Biotoxicity of Water-Soluble UV Photodegradation Products for 10 Typical Gaseous VOCs

**DOI:** 10.3390/ijerph15071520

**Published:** 2018-07-18

**Authors:** Zhuqiu Sun, In-Sun Kang, Qianyuan Wu, Jinying Xi, Hongying Hu

**Affiliations:** 1 Environmental Simulation and Pollution Control State Key Joint Laboratory, School of Environment, Tsinghua University, Beijing 100084, China; szqqh123@163.com (Z.S.); ISKqh123@163.com (I.-S.K.); hhyqh123@163.com (H.H.); 2 State Environmental Protection Key Laboratory of Microorganism Application and Risk Control (SMARC), Graduate School at Shenzhen, Tsinghua University, Shenzhen 518055, China; wqyqh123@163.com

**Keywords:** VOCs, UV photodegradation, biotoxicity, luminescent bacteria, *Daphnia magna*

## Abstract

Ultraviolet (UV) photodegradation is increasingly applied to control volatile organic compounds (VOCs) due to its degradation capabilities for recalcitrant compounds. However, sometimes the UV photodegradation products are also toxic and can affect human health. Here, 10 VOCs at 150~200 ppm in air were treated using a laboratory-scale UV reactor with 185/254 nm irradiation, and the biotoxicity of their off-gas was studied by investigating their off-gas absorption solutions. The CO_2_ increase and VOC decrease were 39~128 ppm and 0~42 ppm, respectively, indicating that the VOCs and their products were mineralized in off-gas absorption solutions. The total organic carbon (TOC) of the absorption solutions are 4~20 mg∙L^−1^. Luminescent bacteria and *Daphnia magna* were used to detect the acute toxicity, and an *umu* assay was used to determine the genotoxic potential. Trichloroethylene showed a highest toxicity to luminescent bacteria, while chlorobenzene had the lowest toxicity. Water-soluble UV photodegradation products for styrene are very toxic to *Daphnia magna*. In the *umu* assay, the genotoxicities of off-gas absorption solutions of trichloroethylene, methylbenzene, ethyl acetate, butyl alcohol, and styrene were 51.26, 77.80, 86.89, 97.20, and 273.62 mg (4-NQO)·L^−1^ respectively. In addition, the analysis of the genotoxicity/TOC and intermediates products indicated that the off-gas absorption solutions of styrene, trichloroethylene, and butyl alcohol contain many highly toxic substances.

## 1. Introduction

Volatile organic compounds (VOCs) are one of the major contributors to air pollution. They are often found in industrial waste gas from chemical manufacturing plants, hazardous sites, and groundwater remediation facilities [1]. They not only contribute to photochemical smog, but also stratospheric ozone depletion and ecological destruction [2,3]. Large-scale remediation technology is urgently needed [3].

VOCs can be removed via thermal oxidation, adsorption, biofiltration, non-thermal plasma, photodegradation, photocatalysis, etc. [4,5,6]. Of these, ultraviolet (UV) photodegradation is effective at oxidizing and mineralizing most VOCs, including many recalcitrant compounds [7]. Photodegradation is more economical and easy to use versus other advanced oxidation processes (AOPs). The core component of the photoreactor for VOC removal is a low-pressure mercury UV lamp, producing strong irradiation with 85% output UV light at 254 nm and 15% output at 185 nm [8]. In these processes, strong oxidizing substances such as ozone and hydroxyl radical react with VOCs to create water and carbon dioxide. This is suitable for waste gas treatment due to the richer oxygen and lower number of radical scavengers in the gas phase [9]. This has attracted attention over the last two decades [10].

However, some previous studies have shown that water-soluble products with higher toxicities than their parent contaminants can be produced during UV photodegradation. Wang et al. reported that gaseous chlorobenzene was converted to compounds with high toxicities [11]. Cantavenera et al. showed some genotoxic products during a paraquat photocatalytic process [12]. Koh et al. also identified significant acute toxicities for the photodegradation products of several chlorinated paraffins [13]. The intermediates of photooxidation often contain many toxic compounds, such as aldehydes and carboxylic and/or dicarboxylic acids [14]. These toxic compounds from the off-gas of UV photodegradation may have both short- and long-term adverse human health effects, including sensory irritation, allergies, sick building syndrome (SBS), decrements in lung function, asthma, and even leukemia—particularly in vulnerable groups, such as children [15].

UV photodegradation is also frequently chosen as a pretreatment step before biotreatment technologies. Hydrophobic and recalcitrant compounds were disposed using the combined UV-biofilter—the combined system offered much better removal capability than the single system [7,11,16,17]. In this process, UV irradiation produces ozone that can enhance biofilter performance and control biomass over the long-term operation [18]. However, the water-soluble products with high toxicities of UV photodegradation have a potential risk for the application of UV photodegradation as a pretreatment tool before subsequent biofiltration. Therefore, the biotoxicity evaluation of water-soluble UV photodegradation products is indispensable for human health and the application of treatment technology. However, most previous work on the photodegradation or UV-biofilter treatment of VOCs has focused on removal efficiency (RE). Less attention has been devoted to the study of biotoxicity of UV photodegradation products for various VOCs based on different assessment methods.

In this work, 10 typical VOCs with a high RE were studied after UV photodegradation. These served as target pollutants, and their products were collected. The biological toxicity of these products was investigated via acute and genetic toxicities of the off-gas material.

## 2. Materials and Methods

### 2.1. Chemicals

The properties of 10 kinds of VOCs, including molecular weight (MW) and boiling point (BP), are shown in Table 1. These hydrocarbons are all frequently used in the industrial sector with enormous amounts of annual consumption. They are also typical gaseous pollutants. UV photodegradation can treat these VOCs and offers excellent performance. All of the chemicals are liquid at room temperature and were obtained from Shanghai Aladdin Biochemical Technology Co., Ltd. (Shanghai, China) with over 99% purity with no further purification.

### 2.2. UV Photodegradation Reactor Design

**Figure 1:**
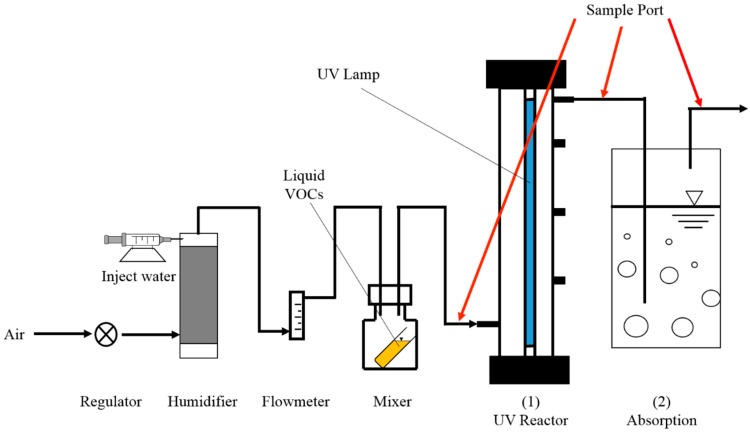
Schematic of the experimental system.

The schematic of the UV photodegradation reactor for VOC removal is shown in Figure 1. The main body of the UV photodegradation reactor was made of a 0.883 L stainless steel cylinder with a diameter of 20 cm and a height of 50 cm. A low-pressure mercury lamp that emitted UV radiation at 254 and 185 nm (Cnlight, ZW23D15Y-Z436, Foshan, China) with a power output of 23 W was inserted into the airtight reactor. The photodegradation experiment used air as the carrier gas for each VOC. A syringe pump (LongerPump, TJ-3A, Baoding, China) fed water into a 3-L tank and controlled the relative humidity of the air. The target concentration of gaseous VOCs was obtained by blowing saturated steam from a 500-mL bottle containing liquid VOCs.

The absorption reactor was assembled behind the UV reactor. The absorption reactor was made of glass and had an inner diameter of 53.2 mm and a height of 240 mm. This resulted in a total volume of 0.533 L. The 0.4 L of distilled water was added into the absorption reactor before each absorption operation. The off-gas was injected into the water through a glass tube with four small holes on the top of the tube with a diameter of 1.5 mm. The distance between the gas outlet and the water level was 155 mm, and the residence time of off-gas in the absorption reactor was 21 s. The air was continuously fed into the experimental system at a flow rate of 0.06 m^3^·h^−1^. To simulate the actual working state, the gas temperature and relative humidity of the air was maintained at 23 ± 2 °C (average room temperature) and 70 ± 5% (average indoor humidity), respectively. The inlet VOCs concentrations for each VOCs in air were controlled at 150~200 ppm (all of the selected VOCs can be effectively treated by UV photodegradation reactor). The off-gas was continuously fed into the distilled water in the absorption reactor when the UV lamp was running.

### 2.3. Experimental Procedure

The flow rate and the inlet concentration of VOC gases were adjusted to 0.06 m^3^·h^−1^ and 150~200 ppm, respectively, before starting the UV lamp. The gas samples were collected from the inlet and outlet of the UV reactor after 30 min of operation. Between each experiment for different VOCs, the reactor and the pipeline were cleaned to avoid cross contamination. Ten VOCs were degraded by UV photodegradation. Then, the off-gas containing VOCs and their degradation products were bubbled into water. The VOCs and their degradation products dissolve in the water to form off-gas absorption solutions.

The total organic carbon (TOC), total nitrogen (TN), and total mass of carbons (MC) of the absorption solutions were measured first. Each experimental dataset represents at least 3 replicates. Then, we studied the toxicities including the acute toxicities (tested by luminescent bacteria and *Daphnia magna*) and genotoxicity of the absorption liquid via *umu* testing. Finally, the intermediate products of highly biotoxicity VOCs were detected by gas chromatography-mass spectrometry (GC-MS). All of the experiments were performed in duplicate.

### 2.4. Analytical Methods

The TOC and TN were measured with a TOC analyzer (TOC-VCPH, Shimadzu, Japan) with a TN measuring unit (TNM-1, Shimadzu, Japan).

Luminescent bacteria are often used for toxicity testing in environmental or industrial settings. They offer high sensitivity, simple operation, and low costs [19,20,21]. The luminescence of these bacteria is a normal metabolic activity, and the luminescent intensity is constant under normal conditions [22]. However, the luminescence can decrease when the bacteria are stressed by toxicants. This signal change correlates with the concentration of the samples and the toxicity of the samples. Luminescent bacteria testing can use the standardized protocol GB/T 15441-1995 measured by a multi-detection microplate reader system SpectraMax M5 (Molecular Devices Corporation, San Jose, CA, USA).

*Daphnia magna* was used to investigate acute and chronic toxicity in Delupis et al. [23,24]. *Daphnia magna* was put into samples (original, 1/2 dilution, 1/4 dilution, 1/8 dilution, 1/16 dilution), and the acute toxicity was studied over the next 24 h. The concentrations were recorded when 50% of *Daphnia magna* was dead or showed inhibition as the reflecting of biotoxicity value (GB/T 16125-2012).

The *umu* assay is an effective method to determine genotoxic potential; it was introduced by Oda et al. [25]. When bacteria are subjected to DNA damage about 30 genes are coordinately induced, known as “SOS response”. The *umu* assay studies environmental mutagens that induce DNA damage. The β-galactosidase would be expressed and induced by *umu* C. The β-galactosidase activity could be characteristic of genotoxic activity of the environmental mutagens [26]. The *umu* assay was applied for dynamic analysis and environmental quality assessment. It is simple, efficient, and sensitive. The genotoxicity of the concentrated samples was evaluated with the SOS/*umu* test based on Salmonella typhimurium TA1535/pSK1002 without S9 activation in triplicates (ISO 13829).

The intermediate products of highly biotoxicity VOCs were detected by GC-MS (Mars-400, Focused Photonics Inc., Hangzhou, Zhejiang Province, China) with a capillary column (5 m × 0.1 mm × 0.4 μm, DB-5 ms). The temperatures of the injector, transfer line and MS detector were 150, 150, and 70 °C, respectively. The total flow of the carrier gas (He with 99.999% purity) was measured to be 40 mL/min at a split ratio 1:200. The column temperature was programmed as follows: hold for 0.5 min at 50 °C, 50~70 °C at 20 °C/min, 70~220 °C at 60 °C/min, and hold for 0.5 min at 220 °C.

## 3. Results and Discussion

### 3.1. TOC and TN of Off-Gas Absorption Solutions

The TOC and TN of the off-gas absorption solutions for the 10 VOCs after UV photodegradation reactors are shown in Table 2. The TOC of the absorption solutions are 4~20 mg∙L^−1^, and the relative total mass of the organic carbon is 1.7~7.8 mg for the 10 VOCs. The original total mass of the carbon in VOCs injected into the UV reactor (MC) was estimated to be about 10 to 45 mg. More than 80% of the selected VOCs were removed by the UV reactor in toxicity tests, and most TOC in the absorption solutions contain organic intermediates of UV photodegradation. This suggests that 6~35% VOCs were transferred to other organics after UV irradiation.

Ethyl acetate and styrene were particularly high at 19.6 and 18.53 mg·L^−1^, respectively, indicating that these two VOCs were minimally degraded by UV photodegradation [27]. However, propionaldehyde, tetrahydrofuran, and chlorobenzene show a comparatively low concentration of TOC at 4.18, 4.31, and 4.96 mg·L^−1^, respectively. This shows that UV photodegradation is suitable for these VOCs.

TN in 5 of 10 VOCs was detected in this work, indicating that the nitrogen in air could be oxidized by UV reaction and absorbed in the aqueous phase. This might be because some photocatalytic reactions occurred, using oxidized metal on stainless steel cylinder or some products as catalyst, and the nitrogen oxidation using photocatalysis was presented in previous studies [28]. These VOCs were degraded sequentially in the absorption reactor, and CO_2_ concentration significantly increased. This might be because the residual VOCs or intermediates were oxidized by ozone from UV photodegradation.

### 3.2. Acute Toxicity for Luminescent Bacterium

**Figure 2:**
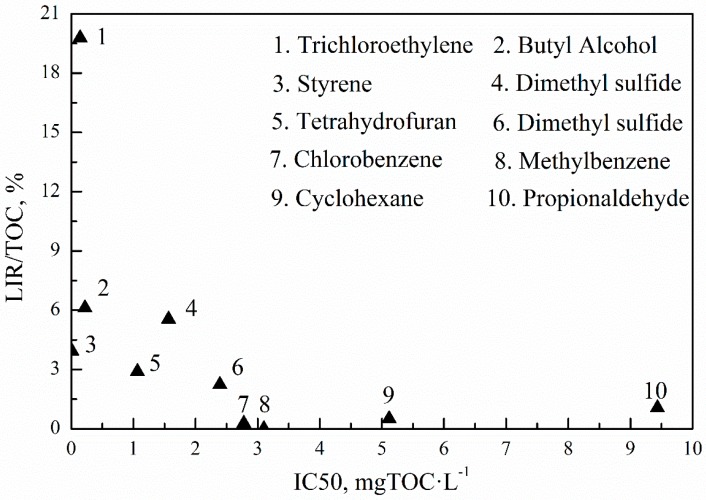
Luminescent inhibition ratio (LIR)/TOC and half maximal inhibitory concentration (IC50) of luminescent bacterium for 10 VOCs, including off-gas absorption solutions.

The luminescent inhibition ratio (LIR) represents the acute toxicity of off-gas absorption solutions for the 10 VOCs listed in Figure 2. The intensities of the bioluminescence light of the reference (IR) and the sample (IS), respectively, were determined. The luminescent inhibition ratio (LIR) was calculated according to Equation (1):

(1) $$\mathrm{LIR}=\frac{Ir-Is}{Ir}\times 100\%.$$

The luminescent inhibition of luminescent bacteria was over 90% for each kind of VOCs in primary liquid (the undiluted liquid after gas absorption). It is difficult to assess acute toxicity by luminescent bacteria. In order to enhance differentiation, the primary liquid needs to be diluted using water. The dilution rate was 2, 3, 5, 10, and 100 respectively. The results are shown in Table 3. The luminescent inhibition was also over 90% when the dilution rate doubled. It was 0% with 100-fold dilution. All of the specimens were easily identified when diluted 10:1. Trichloroethylene showed the highest luminescent inhibition when diluted 10:1, and the luminescent inhibition of chlorobenzene was almost 0%.

The LIR/TOC and half maximal inhibitory concentration (IC50) of off-gas adsorption solutions for 10 VOCs are represented in Figure 2. The high LIR/TOC and low IC50 both showed high acute toxicity for luminescent bacterium. Chlorobenzene, methylbenzene, cyclohexane, and propionaldehyde showed a low acute toxicity for luminescent bacterium. The production should be low or non-toxic for luminescent bacteria. The LIR/TOC of styrene, trichloroethylene, and butyl alcohol are higher than other VOCs and their IC50 are also very low indicating that enormous amounts of toxic chemicals were produced after UV photodegradation [29].

### 3.3. Acute Toxicity for Daphnia Magna

**Figure 3:**
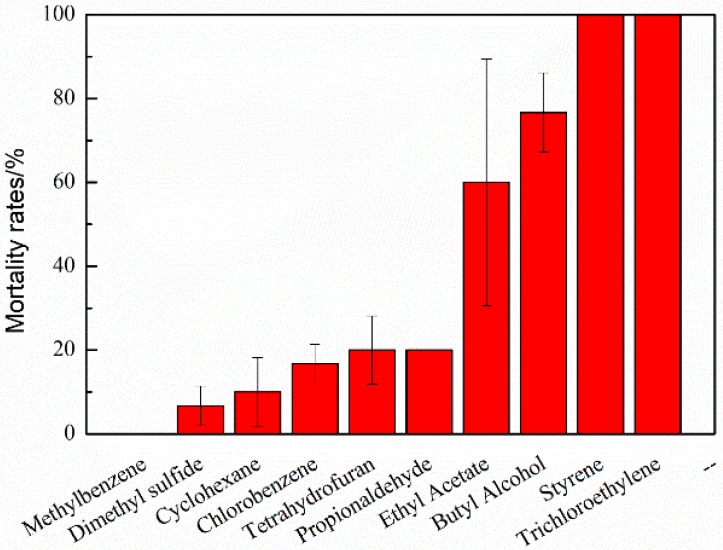
Mortality rates of *Daphnia magna* for 10 VOCs.

Mortality rates of *Daphnia magna* are presented in Figure 3. There are six types of off-gas absorption solutions that are less toxic to *Daphnia magna*. *Daphnia magna*’s mortality was always less than 50%, especially for methylbenzene with hardly no impact on the bacteria after UV photodegradation. The products of ethyl acetate, butyl alcohol, styrene, and trichloroethylene were obviously toxic to *Daphnia magna*, indicating that these four VOCs need more degradation using other technologies.

### 3.4. Genotoxicity

**Figure 4:**
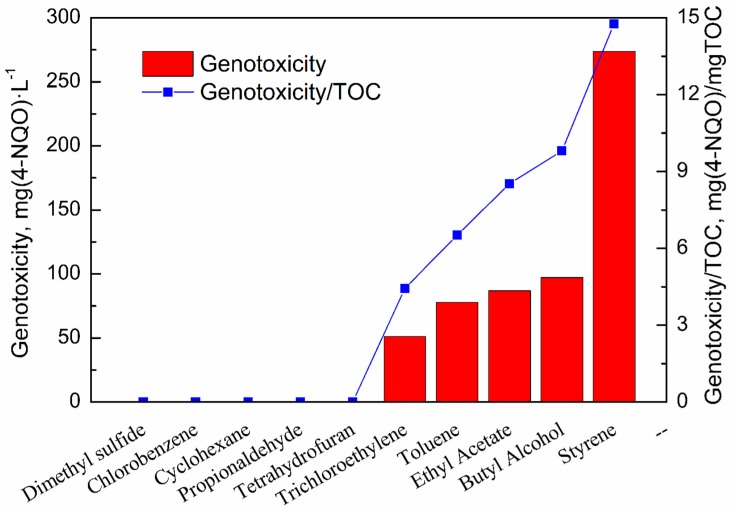
Genotoxicity and genotoxicity/TOC of the off-gas absorption solutions for 10 VOCs.

The genotoxicity analysis of the samples is shown in Figure 4. The absorption solutions containing the off-gas of dimethyl sulfide, chlorobenzene, cyclohexane, propionaldehyde, and tetrahydrofuran did not affect β-galactosidase activity. They could not induce DNA damage [26]. In other words, the genotoxicity of the off-gas absorption solutions for these VOCs is low. However, the off-gas absorption solutions containing the degradation products of trichloroethylene, methylbenzene, ethyl acetate, butyl alcohol, and styrene were genotoxic: 51.26, 77.80, 86.89, 97.20, and 273.62 mg (4-NQO)·L^−1^, respectively. Above all, the UV degradation products of styrene have the highest genotoxicity, similar to acute toxicity testing.

The genotoxicity of VOC byproducts was also assessed by the genotoxicity/TOC (Figure 4). Although the TOC of absorption solutions for methylbenzene, tetrahydrofuran, and styrene are lower than 15 mg·L^−1^, the genotoxicity/TOC is still high, indicating that the off-gas absorption solution of methylbenzene, tetrahydrofuran, and styrene contains many highly genotoxic substances. The TOC and genotoxicity of the absorption solutions for chlorobenzene and cyclohexane are both so low that they cannot be detected, indicating that they can be better degraded by UV. In addition, the off-gas absorption solution of propionaldehyde has high TOC but has low genotoxicity. This result shows that the products of propionaldehyde are mostly nontoxic organics. The genotoxicity of the secondary effluent water from sewage treatment plant is much higher (1–2 orders of magnitudes) [30], as is the genotoxicity of trichloroethylene, methylbenzene, butyl alcohol, and styrene. The genotoxicity/TOC data demonstrate that trichloroethylene, methylbenzene, ethyl acetate, butyl alcohol, and styrene can be degraded by UV photodegradation, but other treatment processes are needed.

### 3.5. Intermediate Production

**Figure 5:**
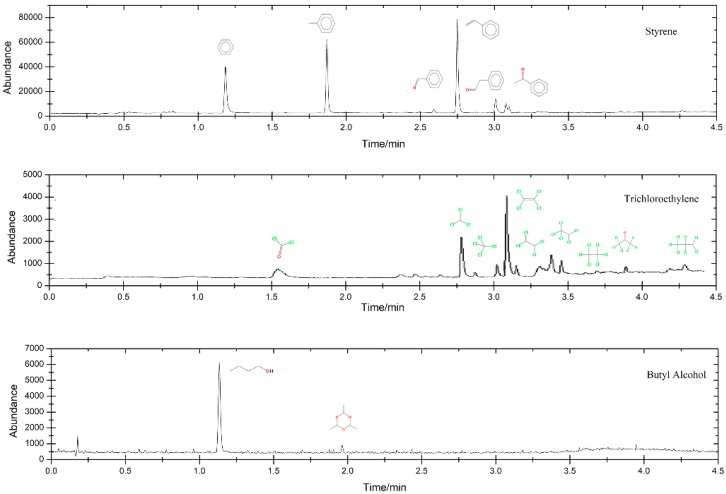
Spectrograms of styrene and butyl alcohol intermediates/byproducts.

The intermediates of styrene, trichlorethylene, and butyl alcohol were analyzed by GC-MS due to their high biotoxicity. The spectrograms are presented in Figure 5. Many aromatic compounds were identified in the off-gases of styrene such as benzene, toluene, benzaldehyde, benzene acetaldehyde, and phenyl methyl ketone indicating that styrene was basically not oxidized for ring opening. The intermediates increased the biotoxicity of the off-gas absorption solution. Many organic chlorides, including phosgene, carbon tetrachloride, tetrachloroethylene, 1,1,2,2-tetrachloroethane, pentachloroethane, hexachloroacetone, heptachloropropane, and hexachloroethane, were discovered in the UV photodegradation off gas of trichlorethylene; they are all hypertoxic for biology. Paraldehyde is the intermediate produced by butyl alcohol. It might be one of the main causes of the high biotoxicity. Therefore, more work is needed to study in order to remove these toxic species.

## 4. Conclusions

The TOC and TN of 10 VOCs demonstrates that some of the VOCs were transferred to other water-soluble organics after UV photodegradation. The CO_2_ concentration changes and VOC removal indicates that residual VOCs or their intermediates were oxidized in the absorption reactor.

Styrene, trichlorethylene, and butyl alcohol had a higher toxicity to luminescent bacteria and *Daphnia magna*. Dimethyl sulfide showed a higher toxicity to luminescent bacteria and lower toxicity to *Daphnia magna*. Chlorobenzene, methylbenzene, and cyclohexane had low toxicity to *Daphnia magna* and luminescent bacteria. The *umu* test evaluated genotoxicity and found it in five VOCs. Styrene had the highest toxicity among these.

Chlorobenzene and cyclohexane can be degraded effectively via UV photodegradation, and the off-gas absorption solutions have low biotoxicity. High toxicity intermediates were found in the off-gas of UV photodegradation for styrene, trichlorethylene, and butyl alcohol.

## Figures and Tables

**Table 1:** Properties of the 10 volatile organic compounds (VOCs) used in toxicity experiments.

VOCs	Molecular Formula	MW/(g·mol^−1^)	BP/°C
Propionaldehyde	C_3_H_6_O	58.08	48.0
Trichloroethylene	C_2_HCl_3_	131.39	87.1
Chlorobenzene	C_6_H_5_Cl	112.56	131.0
Dimethyl sulfide	(CH_3_)_2_S	62.13	37.5
Tetrahydrofuran	(CH_2_)_4_O	72.11	66.0
Methylbenzene	C_6_H_5_CH_3_	92.14	111.0
Butyl Alcohol	CH_3_(CH_2_)_3_OH	74.12	117.3
Cyclohexane	C_6_H_12_	84.62	80.7
Styrene	C_6_H_5_CH=CH_2_	104.14	145.2
Ethyl Acetate	CH_3_COOCH_2_CH_3_	88.11	77.2

**Table 2:** Total organic carbon (TOC) and total nitrogen (TN) of off-gas absorption solutions after treatment in the absorption reactor.

VOCs	TOC/(mg·L^−1^)	TN/(mg·L^−1^)	MC/mg	CO_2_ Increase/(ppm)	VOC Decrease/(ppm)
Propionaldehyde	4.18	0.37	16.9	97	12
Trichloroethylene	4.31	0	22.5	41	0
Chlorobenzene	4.96	0	33.8	56	7
Dimethyl sulfide	5.79	0.60	11.3	39	8
Tetrahydrofuran	7.87	0.26	11.3	95	8
Methylbenzene	9.13	0.15	39.4	128	6
Butyl Alcohol	9.92	0	22.5	45	18
Cyclohexane	16.55	0	33.8	81	42
Styrene	18.53	0.50	45.0	41	8
Ethyl Acetate	19.60	0	22.5	72	27

**Table 3:** Luminescent inhibition ratio (LIR) (%) at different dilution rates for VOC off-gas absorption solutions.

VOCs	1	1/2	1/3	1/5	1/10	1/100
Propionaldehyde	94.28	95.79	84.36	37.15	0	0
Trichloroethylene	96.48	95.29	74.19	41.58	2.54	0
Chlorobenzene	98.17	97.73	83.74	39.68	4.44	0
Dimethyl sulfide	100	99.88	87.39	51.14	8.46	0
Tetrahydrofuran	97.52	96.29	92.69	61.39	22.77	0
Methylbenzene	99.28	99.17	94.73	49.84	32.13	0
Butyl Alcohol	94.36	93.89	90.66	80.37	43.82	0
Cyclohexane	99.93	96.85	94.57	90.31	60.69	0.01
Styrene	99.87	96.49	97.49	92.22	72.69	0
Ethyl Acetate	100	100	99.44	100	85.28	0.02
